# Validation of the Turkish Version of the Eating Attitudes Test-7

**DOI:** 10.3390/nu18142344

**Published:** 2026-07-17

**Authors:** Gamze Ayakdaş, Meryem Kahrıman, Ladan Hajhamidiasl, Yaşar Alp Erol, Selen Köksal, Perim Fatma Türker, Murat Baş

**Affiliations:** Department of Nutrition and Dietetics, Faculty of Health Sciences, Acibadem Mehmet Ali Aydinlar University, Istanbul 34752, Turkey; gamze.ayakdas@acibadem.edu.tr (G.A.); ladan.hajhamidiasl@acibadem.edu.tr (L.H.); alp.erol@acibadem.edu.tr (Y.A.E.); selen.koksal@acibadem.edu.tr (S.K.); perim.turker@acibadem.edu.tr (P.F.T.); murat.bas@acibadem.edu.tr (M.B.)

**Keywords:** Eating Attitudes Test-7, EAT-7, disordered eating attitudes, eating disorders, validity, reliability, Turkish adaptation

## Abstract

**Background**: Disordered eating attitudes are major public health concerns associated with adverse physical, psychological, and social outcomes. Therefore, brief, psychometrically sound screening tools are needed to identify individuals at risk early. The Eating Attitudes Test-7 (EAT-7) is a shortened screening tool developed to assess disordered eating attitudes. However, its Turkish validity and reliability have not been previously examined. Therefore, this study aimed to assess the validity and reliability of the Turkish version of the EAT-7 among Turkish-speaking adults. **Methods**: This methodological validation study was conducted between November 2025 and January 2026. Data were collected online using a snowball sampling technique. After a pilot study with 210 participants, the main study included 1635 adults aged ≥18 years. Exploratory factor analysis was performed with 500 participants, and confirmatory factor analysis was performed with an independent sample of 1135 participants. Internal consistency and construct validity were assessed. Convergent validity was evaluated through correlations with the EAT-26, Eating Disorder Examination Questionnaire-13 (EDE-Q-13), and Depression Anxiety Stress Scale Short Form (DASS-8). **Results**: The Turkish version of the EAT-7 demonstrated excellent internal consistency, with a Cronbach’s alpha coefficient of 0.925. The Kaiser–Meyer–Olkin value was 0.909, and Bartlett’s test results were significant. Exploratory factor analysis supported a single-factor structure explaining 70.133% of the total variance. Confirmatory factor analysis confirmed a good model fit. The total EAT-7 score was positively correlated with the EAT-26, EDE-Q-13, and DASS-8 scores. **Conclusions**: The Turkish version of the EAT-7 is a valid, reliable, brief, and practical screening tool for assessing disordered eating attitudes among Turkish-speaking adults.

## 1. Introduction

Eating disorders are a major public health concern worldwide due to their increasing prevalence. These disorders adversely affect not only physical health but also psychological well-being and social functioning. Eating disorders are associated with anxiety and depression, reduced quality of life, and increased healthcare costs [1]. Early identification and intervention of eating disorders can substantially improve prognosis. However, culturally appropriate, valid, and reliable screening tools are needed for this purpose.

The Eating Attitudes Test (EAT) is one of the most widely used tools for screening anorexia nervosa and other eating disorders [2]. The Eating Attitudes Test was originally developed by Garner and Garfinkel in 1979 as a 40-item scale (EAT-40) and later revised by Garner et al. in 1982 into a shorter 26-item version [3,4]. The validity and reliability of the EAT-26 have been examined across cultures. However, its length may pose challenges in field studies and routine clinical practice. Therefore, shorter versions have been developed in different languages and cultural contexts, and brief forms, such as the EAT-18, EAT-8, and EAT-7, have been introduced [5,6].

Recently, increasing attention has been paid to the EAT-7 as a brief and highly feasible screening tool. Fekih-Romdhane et al. evaluated the EAT-7 in an Arabic-speaking population and reported its validity and reliability. The unidimensional structure of the scale demonstrates strong psychometric properties, with Cronbach’s alpha values ranging from 0.88 to 0.92. In their study, the scale demonstrated high internal consistency, construct validity, and strong discriminant validity [5]. A recent study showed that the EAT-7 preserves its construct validity among patients with first-episode schizophrenia, demonstrates measurement invariance across women and men, and shows significant positive correlations with depression and anxiety [7]. These findings indicate that the EAT-7 is a brief, comprehensible, and feasible screening tool that can be used not only in the general population but also in psychiatric risk groups.

Studies conducted in Turkey have demonstrated the prevalence of eating disorders, particularly among adolescents and young adults. Studies among university students have reported that the risk of eating disorders ranges from 10% to 25%, with higher rates among female students [8,9]. Additionally, studies conducted among adolescents have shown associations between disturbed eating attitudes and anxiety, low self-esteem, and body image concerns [10]. These findings indicate a critically high prevalence of eating disorders among young people in Turkey and underscore the need for early screening and intervention due to their psychosocial consequences.

Disordered eating attitudes have been reported to be an important concern not only among young people but also among middle-aged and older adults in Turkey. Particularly, disturbed eating attitudes have been highly reported among middle-aged women in relation to body weight, body image concerns, and psychosocial factors [11]. Furthermore, eating disorders may follow a lifelong course, are associated with comorbid psychiatric conditions, and pose significant risks in terms of health outcomes, highlighting the importance of early diagnosis and intervention not only for young populations but also for adults [1]. These findings highlight the need to assess and screen disturbed eating attitudes across different age groups at an early stage.

The validity and reliability of the Turkish version of the EAT-26 were previously examined by Ergüney-Okumuş and Sertel-Berk in 2019, who reported that the scale was a valid instrument for assessing eating attitudes [12]. However, a Turkish version of the EAT-7 has not yet been developed. Adapting the EAT-7 into Turkish will contribute to rapid screening in clinical settings and enable a more practical assessment of disturbed eating attitudes among Turkish individuals. Furthermore, this adaptation can address an important gap in the national and international literature.

This study may contribute to the early detection of disordered eating attitudes in Turkey, provide a culturally appropriate brief measurement tool, and offer a strong basis for future scientific research. This study aimed to evaluate the validity and reliability of the Turkish version of the EAT-7 and its usability among Turkish-speaking individuals.

## 2. Materials and Methods

### 2.1. Study Design and Participants

This methodological validation study was conducted between November 2025 and January 2026. Data were collected online through Google Forms distributed via social media platforms using a snowball sampling technique.

This study was conducted in two stages. First, a pilot study was conducted to evaluate the linguistic clarity, comprehensibility, and cultural appropriateness of the translated Turkish version of the EAT-7. Participants were asked to identify any unclear wording or difficulties in understanding the questionnaire items and response options. In addition, preliminary internal consistency reliability was assessed to identify any potentially problematic items before the main validation study. Based on participant feedback and expert evaluation, only minor linguistic refinements were made to improve readability, while no substantive modifications or item deletions were required. Second, the main study examined the psychometric properties of the scale. Separate independent samples were recruited for the pilot and main studies.

The sample size was determined based on recommendations in the psychometric literature, which indicates that the number of participants should be at least 5–10 times the number of scale items [13]. Since the EAT-7 consists of seven items, the pilot study sample size was planned to be 30 times the number of items (*n* = 210). The pilot study included 210 participants. Of the 210 individuals who participated in the pilot study, 33.3% were males, and 66.7% were females. The mean age of the male participants was 28.54 ± 11.03 years. The pilot sample was adequate to represent the target population in terms of basic sociodemographic characteristics. Additionally, a preliminary comparison of responses to Item 2 (“I know the calorie content of the foods I eat”) by gender was conducted (Table 1). The effect size was calculated using Cohen’s r, a commonly used effect size measure in the literature, alongside Cohen’s d, Hedges’ g, and Glass’s Δ. According to Cohen’s criteria, an effect size of <0.20 indicates a small effect, 0.20–0.50 indicates a moderate effect, and ≥0.80 indicates a large effect. However, even smaller effect sizes may be meaningful depending on the research context [14,15].

In the pilot study, the Cronbach’s alpha coefficient of the EAT-7 was 0.909, indicating high internal consistency. Item-total analyses revealed that Cronbach’s alpha values ranged between 0.883 and 0.914, and no item had a corrected item-total correlation <0.30. These findings indicated that all items were well understood and demonstrated satisfactory preliminary performance in the Turkish version. Therefore, no items required deletion or substantial revision before the main psychometric validation study. Therefore, no items were removed from the scale [13,14].

After the pilot study, an a priori power analysis was performed using R software version 4.3.1 (R Core Team, 2025, Vienna, Austria). With an effect size of 0.294, an alpha error of 5%, and a beta error of 10%, the minimum sample size required for the main study was 244 participants [15,16]. Larger independent samples were collected for exploratory factor analysis (EFA) and confirmatory factor analysis (CFA) to strengthen the psychometric analyses. Based on the power analysis, 1635 participants were recruited for the main study. The sample was divided into two separate independent samples for EFA (*n* = 500) and CFA (*n* = 1135).

This study included individuals aged ≥18 years who voluntarily agreed to participate. This study was approved by the Acibadem Mehmet Ali Aydinlar University Medical Research Ethics Committee (approval number 2025-16/605) and conducted in accordance with the ethical principles of the Declaration of Helsinki. All participants were informed about the study’s objectives and procedures, and electronic informed consent was obtained before participation.

The questionnaire consisted of four sections: (1) sociodemographic characteristics and general eating habits, (2) the EAT-7, (3) the Eating Disorder Examination Questionnaire-13 (EDE-Q-13), and (4) the Depression Anxiety Stress Scale Short Form (DASS-8).

### 2.2. Measurements

#### 2.2.1. Eating Attitudes Test-7

The EAT-7 is a short-form instrument consisting of 7 items developed to assess problematic eating attitudes and behaviors. Each item is rated on a 4-point Likert-type scale ranging from 0 (never) to 3 (always), with higher scores indicating greater impairment in eating attitudes and behaviors. In this study, the validity and reliability of the Turkish version of the EAT-7 were evaluated.

Permission to adapt the EAT-7 was obtained from the original authors. The EAT-7 was translated into Turkish using a standard forward–backward translation procedure. First, the original EAT-7 was translated into Turkish by two independent bilingual researchers. Then, the translated version was back-translated into English by two different bilingual experts. The original and back-translated versions were compared, and any discrepancies were resolved through consensus. The prefinal Turkish version was subsequently evaluated by an expert committee consisting of four experts, who assessed the translated items for semantic, conceptual, idiomatic, and cultural equivalence as well as content validity. Consensus among the experts was reached to ensure that the Turkish version retained the conceptual meaning of the original instrument while remaining culturally appropriate for the target population. The prefinal Turkish version was evaluated in a pilot study to assess clarity and comprehensibility. Participant feedback obtained during the pilot study confirmed that the items were understandable and culturally appropriate. Minor linguistic refinements were made to improve readability; however, no changes affecting the conceptual meaning or content of the questionnaire were necessary. Necessary adjustments were made based on participant feedback and expert evaluation to ensure semantic equivalence and linguistic appropriateness for the target population.

#### 2.2.2. Eating Disorder Examination Questionnaire-13

The EDE-Q was originally developed by Fairburn and Beglin in 1994 [17]. The EDE-Q is a self-report instrument that assesses eating behaviors over the previous 28 days. The EDE-Q consists of 28 items comprising four subscales: restraint, shape concern, weight concern, and eating concern.

The short version of the EDE-Q (EDE-Q-13) was developed by Lev-Ari et al. and consists of 13 items covering five subdimensions: eating restraint, shape and weight overevaluation, body dissatisfaction, binge eating, and self-induced vomiting [18]. The Turkish adaptation and validation study of the EDE-Q-13 was conducted by Esin and Ayyıldız. In this study, the EDE-Q-13 was used to evaluate Convergent validity [19].

#### 2.2.3. Depression Anxiety Stress Scale Short Form

The DASS-8 is a valid, reliable, and useful tool for assessing depression, anxiety, and stress symptoms. The scale consists of 8 items covering three subdimensions: depression (3 items), anxiety (3 items), and stress (2 items). Items are rated on a four-point Likert-type scale, with higher scores indicating greater psychological distress. The validity and reliability of the original scale were established by Ali et al., whereas those of the Turkish version were established by Türk et al. In this study, the DASS-8 was used to assess convergent validity with psychological distress measures [20,21].

### 2.3. Statistical Analysis

All statistical analyses were performed using SPSS version 27 (IBM Inc., Chicago, IL, USA) and R version 3.6.1 (R Core Team, Vienna, Austria). Categorical variables were presented as frequency and percentage, whereas continuous variables were presented as mean ± standard deviation (SD), median, minimum, and maximum. Data normality was assessed using the Shapiro–Wilk test. The internal consistency of the scale was assessed using Cronbach’s alpha coefficient. A Cronbach’s alpha value of >0.80 was considered indicative of high reliability [14,22]. The adequacy of the sample size for factor analyses was examined using the Kaiser–Meyer–Olkin (KMO) test, and the suitability of the data for factor analyses was determined using Bartlett’s test of sphericity.

EFA was performed using principal axis factoring as the extraction method to examine the factor structure of the scale; principal component analysis (PCA) was additionally used as a preliminary data-reduction step to inspect eigenvalues and the overall variance structure prior to EFA, and the two techniques were treated as distinct procedures rather than as equivalents. Items with factor loadings < 0.30 were considered inadequate and excluded from the analysis. Varimax rotation was initially specified in the analysis plan to allow for the possibility of a multi-factor solution; however, because the EFA converged on a single-factor structure, rotation had no mathematical effect on the final solution and is reported here only for methodological transparency. CFA was performed using the maximum likelihood estimation method to evaluate construct validity. Model fit was assessed using the following indices: root mean square error of approximation (RMSEA), normed fit index (NFI), comparative fit index (CFI), goodness-of-fit index (GFI), adjusted goodness-of-fit index (AGFI), and standardized root mean square residual (SRMR), with RMSEA ≤ 0.08, NFI ≥ 0.90, CFI ≥ 0.90, GFI ≥ 0.85, AGFI ≥ 0.85, and SRMR < 0.10 considered reasonable. Convergent validity was assessed through factor loadings and relationships with theoretically related scales [23]. Convergent validity was assessed by examining correlations between the EAT-7 and the EDE-Q-13 and DASS-8 using Pearson’s product–moment correlation coefficient. Correlation coefficients were interpreted as follows: very weak (<0.20), weak (0.20–0.39), moderate (0.40–0.59), strong (0.60–0.79), and very strong (≥0.80) [24].

## 3. Results

### 3.1. Reliability Analysis of the EAT-7

The EAT-7, consisting of 7 items, had a Cronbach’s alpha value of 0.925, indicating high reliability. Examination of the item-total statistics revealed that the Cronbach’s alpha values ranged from 0.901 to 0.927, indicating that no items needed to be removed from the scale (Table 2).

### 3.2. Validity Analysis of the EAT-7

The KMO value of the EAT-7 was 0.909, indicating that the sample size was adequate. Bartlett’s sphericity test chi-square value was χ^2^ = 3007.047, and the *p*-value was <0.001, indicating that the dataset was suitable for EFA (Table 3).

### 3.3. Descriptive Statistics

A total of 500 participants were included in the EFA, and 1135 were included in the CFA. Of the 500 individuals included in EFA, 30% were men, and 70% were women. The mean age was 30.92 ± 11.31 years. According to the body mass index (BMI) classification, 52.4% of the participants were in the normal category. Of the 1135 participants included in the CFA, 38.7% were men, and 61.3% were women. Their mean age was 32.31 ± 11.99 years. According to the BMI classification, 53.7% of the participants were in the normal category (Supplementary Table S1).

### 3.4. EFA

The EFA results revealed a single-factor structure with factor loadings >0.30 and eigenvalues > 1, explaining 70.133% of the total variance. The preliminary PCA, conducted to inspect the scale’s underlying variance structure before extraction, similarly showed no items with factor loadings <0.30, and no items had a difference of at least 0.10 between two low-loading factors. Therefore, no items were removed from the scale. Based on these results, the EAT-7 is a 4-point Likert-type scale with 7 items and a single-factor structure, with a total score ranging from 0 to 21 (Table 4).

### 3.5. CFA

**Figure 1:**
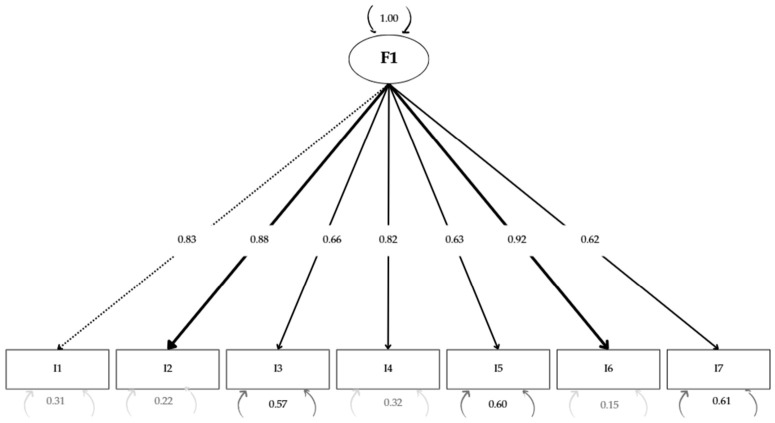
The CFA model of the study.

CFA was used to confirm the single-factor structure of the EAT-7 revealed by the EFA. The criterion values were met in the initial CFA model. No items had factor loadings <0.3. Therefore, no items were removed from the scale (Figure 1).

The structural equation model fit values obtained using the CFA were as follows: χ^2^/df = 1.768, RMSEA = 0.026, NFI = 0.989, CFI = 0.995, SRMR = 0.051, GFI = 0.993, and AGFI = 0.987. The obtained data were within the threshold values, indicating that the model has a good fit index (Table 5).

### 3.6. Relationship Between the EAT-7, EAT-26, EDE-Q-13, and DASS-8

A statistically significant, positive, weak correlation was observed between the total EAT-7 and EDE-Q-13 scores (r = 0.233; *p* < 0.001). Similarly, a statistically significant, positive, and strong correlation was observed between the total EAT-7 and EAT-26 scores (r = 0.755; *p* < 0.001). Furthermore, a statistically significant, positive, but very weak correlation was observed between the total EAT-7 and DASS-8 scores (r = 0.177; *p* < 0.001). These significant positive correlations with theoretically related measures provide evidence supporting the convergent validity of the EAT-7 (Table 6).

## 4. Discussion

This study evaluated the psychometric properties of the Turkish version of the EAT-7. The findings indicate that the scale is a valid and reliable measurement tool in the Turkish population. Eating disorders and disordered eating attitudes encompass a broad pattern of behaviors, including restrictive eating, binge eating, compensatory behaviors, and excessive preoccupation with body weight and shape, and can have significant adverse effects on psychological, physical, and social health [25]. Therefore, developing short, practical screening tools with strong psychometric properties is of great importance, particularly in community-based studies, for early risk identification, planning preventive interventions, and practical data collection in large samples [5]. Recently, short-form scales have been increasingly preferred in psychometric research because they offer a low respondent burden, can be administered quickly, and are easy to use in larger populations [26,27].

In this study, the Cronbach’s alpha coefficient for the EAT-7 was 0.925, indicating very high internal consistency. A Cronbach’s alpha coefficient of >0.90 has been reported to indicate excellent internal consistency [28]. The fact that the scale exhibited a high Cronbach’s alpha coefficient despite consisting of only seven items indicates that it maintains its conceptual integrity despite its short form. Although very high internal consistency coefficients may raise concerns about substantial overlap among items and potential redundancy, the corrected item–total correlations were consistently high, and deletion of individual items did not meaningfully improve the overall Cronbach’s alpha coefficient [29,30]. Thus, while some degree of item overlap cannot be entirely excluded, the item-level findings did not indicate a clearly redundant item whose removal would improve internal consistency [23]. Considering the brief seven-item structure of the EAT-7, these findings suggest a reasonable balance between brevity and coherent assessment of the underlying construct.

The reliability findings obtained in this study are consistent with those of the original validation study of the Arabic version of the EAT-7. Fekih-Romdhane et al. reported a Cronbach’s alpha coefficient of >0.90 for the EAT-7 in a community-based adult sample and reported that the scale demonstrated strong internal consistency despite its short form [5]. Additionally, a more recent clinical validation study conducted among patients with schizophrenia reported a Cronbach’s alpha coefficient of 0.88 [7]. The higher Cronbach’s alpha coefficient observed in this study may be due to community-based sampling, larger sample size, and higher homogeneity of the nonclinical population.

The construct validity analysis yielded a KMO value of 0.909, indicating excellent sample adequacy. Furthermore, Bartlett’s sphericity test showed significant results (χ^2^ = 3007.047; *p* < 0.001), confirming that the dataset is suitable for factor analysis [31]. The EFA results indicated that the scale exhibited a unifactorial structure, explaining 70.133% of the total variance. In social sciences, an explained variance ratio > 60% is considered an indicator of strong construct validity [23]. Furthermore, the factor loadings of all items were high, indicating that they strongly represent the common structure. These findings are consistent with those of the original validation study of the EAT-7 conducted by Fekih-Romdhane et al. Factor analyses supported the scale’s unidimensional structure, explaining 67.62% of the total variance [5]. Similarly, the unidimensional structure has been confirmed in the German short EAT forms [32].

The CFA results support the single-factor structure obtained in the EFA. The model fit indices obtained (χ^2^/df = 1.768; RMSEA = 0.026; CFI = 0.995; NFI = 0.989; GFI = 0.993; AGFI = 0.987) fall within the recommended threshold values, indicating that the model fits quite well [33,34]. Particularly, the low RMSEA and high CFI indicate strong model fit. These findings indicate that the Turkish version of the EAT-7 successfully represents the construct it aims to measure. The higher RMSEA (0.157) observed in the schizophrenia sample may be due to the clinical sample structure and the burden of psychopathology [7].

In this study, the total EAT-7 score was positively correlated with the total EAT-26 and EDE-Q-13 scores, indicating the scale’s convergent validity. The EAT-26 is a widely used tool for assessing the risk of eating disorders and possesses robust psychometric properties [4]. Therefore, the high correlation observed between the short and long forms indicates that the EAT-7 can successfully assess a similar structure despite its shorter form. Furthermore, the correlations between the EAT-7 and EDE-Q-13 indicate that the EAT-7 reflects not only general eating attitudes but also clinically meaningful behavioral patterns, such as body shape concerns, weight concerns, and restrictive eating [17,18]. The stronger correlation between the EAT-7 and EAT-26 may be because both scales assess eating behaviors and disordered eating attitudes. Conversely, the relatively lower correlation between the EAT-7 and EDE-Q-13 may be because the EDE-Q-13 includes broader clinical components, such as body shape concerns, binge eating, and compensatory behaviors.

In this study, statistically significant positive correlations were observed between the EAT-7 total score and the total DASS-8 score and its subscale scores. Disordered eating attitudes have been reported to be associated with depression, anxiety, and psychological stress [25,35]. Particularly, emotional eating and restrictive eating behaviors are associated with emotion regulation processes [36]. Chronic stress may disrupt appetite regulation by affecting cortisol levels by the hypothalamic–pituitary–adrenal axis and may increase emotional eating behaviors [37]. Therefore, the positive relationship between the EAT-7 and DASS-8 scores in this study supports the theoretical relationship between disordered eating behaviors and psychological stress. Similarly, previous EAT-7 studies have reported positive correlations with anxiety and depression symptoms [5,7]. The weak correlation observed with the DASS-8 may partly reflect the distinct constructs assessed by the two instruments. The DASS-8 primarily captures general psychological distress and symptoms of depression, anxiety, and stress, whereas the EAT-7 specifically focuses on disordered eating attitudes and behaviors [5,38]. Accordingly, the low magnitude of the correlation may suggest that the Turkish EAT-7 is more closely related to eating-specific psychopathology than to general psychological distress. This interpretation is also consistent with the substantially stronger correlation observed between the EAT-7 and EAT-26 in our study. However, this finding should be interpreted cautiously and requires further evaluation in clinical samples.

The single-factor structure of the EAT-7offers significant advantages in clinical practice. The scale’s ease of interpretation based on the total score allows rapid assessment in primary care settings, screening studies conducted among university students, and community-based studies. In Türkiye, the EAT-7 could be incorporated into initial assessments in family health centers and routine preventive screenings conducted by university health units. Individuals with elevated scores could then be referred for a more comprehensive evaluation by dietitians, psychiatrists, psychologists, or other relevant healthcare professionals. This stepwise use may support earlier identification of disordered eating attitudes, particularly among young adults, while avoiding the use of the scale as a stand-alone diagnostic instrument [39]. Additionally, its short administration time and low respondent burden can enhance participant compliance and reduce missing data. An important consideration for clinical use is the absence of an established cut-off score for the EAT-7. Although higher scores indicate greater disordered eating attitudes, the current study was not designed to determine a diagnostic or risk threshold. Establishing a Turkish-specific cut-off will require studies comparing EAT-7 scores with clinical diagnoses or an appropriate reference standard and evaluating diagnostic accuracy using receiver operating characteristic analyses. Therefore, EAT-7 scores should currently be interpreted as an indicator of the severity of disordered eating attitudes rather than as a diagnostic threshold. Given the limited availability of short-form eating attitude scales in Turkish, the EAT-7 offers a practical and reliable tool for use in the Turkish population, representing a significant contribution.

### Strengths and Limitations

This study has significant strengths. First, this study used a large sample size, enhancing the reliability of the findings. Although the EAT-7 consists of only seven items, its high internal consistency confirms the scale’s strong psychometric properties. Additionally, the scale’s short and practical structure offers significant advantages for rapid data collection in clinical settings and community-based screening studies. However, this study has some limitations. Data were collected using self-report scales, which may introduce social desirability and recall bias. Furthermore, this study was conducted on a community-based, nonclinical sample, which may limit the generalizability of the findings to clinical populations. Test–retest reliability could not be assessed due to the study’s cross-sectional design. Finally, participants were recruited using convenience and snowball sampling through social media platforms. This online recruitment approach may have introduced selection bias by favoring younger and more digitally literate individuals, thereby limiting the generalizability of the findings to the broader Turkish population.

## 5. Conclusions

The early identification of unhealthy eating behaviors is crucial for preventing eating disorders and identifying individuals at risk. Therefore, short, practical, and psychometrically robust screening tools are needed. The results of this study support the validity and reliability of the Turkish version of the EAT-7. Despite its short structure, the scale demonstrates strong internal consistency and construct validity, indicating that it can be used as a practical screening tool for disturbed eating attitudes. Furthermore, given its short and practical structure, the EAT-7 may be useful in community-based studies and clinical settings that require rapid assessment. However, further studies are needed to support the psychometric properties of the scale across different age groups and clinical samples. In particular, future research should validate the Turkish EAT-7 in individuals with clinically diagnosed eating disorders, examine its test–retest reliability, and determine clinically meaningful cut-off scores using diagnostic accuracy analyses. Such studies would clarify its usefulness for identifying individuals at increased risk in clinical settings.

## Figures and Tables

**Table 1:** Comparison of responses to Item 2 (“I know the calorie content of the foods I eat”) by gender in the pilot sample.

	Gender	Mean±SD	t	*p*
Item M2—“I know the calorie conctent of the foods I eat people”	Men	0.21 ± 0.56	−2.119	0.035 *
	Women	0.42 ± 0.84		

t: Independent samples *t*-test. * *p* < 0.05.

**Table 2:** Cronbach’s alpha coefficient and item-total statistics of the EAT-7 scores.

Cronbach’s Alpha Value of EAT-7 Scores			N (Item)	
0.925			7	
	**Scale Mean Score When Item Is Deleted**	**Mean Variance When Item Is Deleted**	**Corrected Item-Total Correlation**	**Cronbach’s Alpha When the Item Is Deleted**
I1	2.4580	17.700	0.814	0.909
I2	2.4740	17.601	0.847	0.906
I3	2.4080	17.969	0.723	0.918
I4	2.4140	17.702	0.751	0.915
I5	2.3580	18.395	0.635	0.927
I6	2.4780	17.388	0.906	0.901
I7	2.3900	17.777	0.708	0.920

**Table 3:** KMO and Bartlett’s test results.

KMO and Bartlett’s Test		
Kaiser-Meyer-Olkin Measure of Sampling Adequacy		0.909
Bartlett’s Test of Sphericity	Approx. Chi-Square	3007.047
	df	21
	Sig.	<0.001 ***

*** *p* < 0.001.

**Table 4:** Factors of the scale identified by the EFA.

	EAT-7 Total
I6	0.943
I2	0.904
I1	0.881
I4	0.830
I3	0.788
I7	0.780
I5	0.712
EVR	70.133
Eigenvalue	4.909

I: item; EVR: explained variance rate.

**Table 5:** Fit indices of the scale.

Fit Index	Threshold Values	Analysis Results
Degrees of Freedom	-	14
Chi-Square/sd	0 ≤ Chi-Square/sd ≤ 2	1.768
RMSEA	RMSA ≤ 0.08	0.026
NFI	0.90 ≤ NFI ≤ 1.00	0.989
CFI	0.90 ≤ CFI ≤ 1.00	0.995
SRMR	SRMR < 0.08	0.051
GFI	0.85 ≤ GFI ≤ 1.00	0.993
AGFI	0.85 ≤ AGFI ≤ 1.00	0.987

RMSEA: Root Mean Square Error of Approximation, NFI: Normed Fit Index, CFI: Comparative Fit Index, SRMR: Standardized Root Mean Square Residual, GFI: Goodness of Fit Index, AGFI: Adjusted Goodness of Fit Index.

**Table 6:** Correlation coefficients between the EAT-7 total score and the EAT-26, EDE-Q-13, and DASS-8 scores.

		1	2	3	4
EAT-26 Total	r	1.000			
	*p*	-			
EDE-Q Total	r	0.266	1.000	0.833	0.788
	*p*	<0.001 ***	-	<0.001 ***	<0.001 ***
DASS-8 Total	r	0.198	0.326	1.000	0.268
	*p*	<0.001 ***	<0.001 ***	-	<0.001 ***
EAT-7 Total	r	0.755	0.233	0.177	1.000
	*p*	<0.001 ***	<0.001 ***	<0.001 ***	-

EAT-26: Eating Attitudes Test-26, EDE-Q: Eating Disorder Examination Questionnaire, DASS: Depression Anxiety Stress Scales, EAT-7: Eating Attitudes Test-7. r: Pearson Correlation Coefficient. *** *p* < 0.001.

## Data Availability

The data presented in this study are available from the corresponding author upon reasonable request. The data are not publicly available due to privacy or ethical restrictions.

## Supplementary Materials

The following supporting information can be downloaded at: https://www.mdpi.com/article/10.3390/nu18142344/s1, Supplementary Table S1: Descriptive findings of participants who were included in the EFA and CFA.

## Abbreviations

The following abbreviations are used in this manuscript:

AGFI	Adjusted goodness-of-fit index
BMI	Body mass index
CFA	Confirmatory factor analysis
CFI	Comparative fit index
DASS-8	Depression Anxiety Stress Scale-8
EAT	Eating Attitudes Test
EAT-7	Eating Attitudes Test-7
EAT-26	Eating Attitudes Test-26
EDE-Q	Eating Disorder Examination Questionnaire
EDE-Q-13	Eating Disorder Examination Questionnaire-13
EFA	Exploratory factor analysis
GFI	Goodness-of-fit index
KMO	Kaiser–Meyer–Olkin
NFI	Normed fit index
PCA	Principal component analysis
RMSEA	Root mean square error of approximation
SD	Standard deviation
SPSS	Statistical Package for the Social Sciences
SRMR	Standardized root mean square residual
