# Supplementary material for: Validation of the Turkish Version of the Eating Attitudes Test-7
Source: Nutrients. 2026 Jul 17;18(14):2344. doi: 10.3390/nu18142344 (PMC13415415; doi:10.3390/nu18142344)
Supplement: Supplementary file 1 [file nutrients-18-02344-s001.zip › nutrients-4411116-supplementary.pdf]

Supplementary Table S1. Descriptive findings of participants who were included in the EFA and CFA

	EFA						CFA					
	**Men (n = 150)**		**Women (n = 350)**		**Total (n = 500)**		**Men (n = 439)**		**Women (n = 696)**		**Total (n = 1135)**	
	n	%	n	%	n	%	n	%	n	%	n	%
**Age (year) ($\bar{X} \pm SD$)**	31.85 ± 12.51		30.52 ± 10.75		30.92 ± 11.31		33.16 ± 12.63		31.77 ± 11.54		32.31 ± 11.99	
**Marital status**												
Married	43	28.7	102	29.1	145	29.0	149	33.9	224	32.2	373	32.9
Single	107	71.3	248	70.9	355	71.0	290	66.1	472	67.8	762	67.1
**Educational status**												
Primary school	1	0.7	10	2.9	11	2.2	8	1.8	17	2.4	25	2.2
Middle school	2	1.3	8	2.3	10	2.0	12	2.7	15	2.2	27	2.4
High school	34	22.7	87	24.9	121	24.2	118	26.9	209	30.0	327	28.8
Undergraduate/associate degree	98	65.3	223	63.7	321	64.2	262	59.7	418	60.1	680	59.9
Postgraduate (master's/doctorate)	15	10.0	22	6.3	37	7.4	39	8.9	37	5.3	76	6.7
**Experiencing binge eating episodes**												
Never	72	48.0	136	38.9	208	41.6	200	45.6	227	32.6	427	37.6
Once a month or less	40	26.7	123	35.1	163	32.6	134	30.5	259	37.2	393	34.6
2-3 times a month	24	16.0	50	14.3	74	14.8	62	14.1	117	16.8	179	15.8
Once a week	8	5.3	17	4.9	25	5.0	23	5.2	46	6.6	69	6.1
2–6 times a week	4	2.7	18	5.1	22	4.4	14	3.2	29	4.2	43	3.8

Once a day or more	2	1.3	6	1.7	8	1.6	6	1.4	18	2.6	24	2.1
**Vomiting as a means of monitoring weight and body shape**												
Never	137	91.3	304	86.9	441	88.2	408	92.9	615	88.4	1023	90.1
Once a month or less	8	5.3	31	8.9	39	7.8	21	4.8	51	7.3	72	6.3
2-3 times a month	2	1.3	9	2.6	11	2.2	7	1.6	14	2.0	21	1.9
Once a week	3	2.0	3	0.9	6	1.2	2	0.5	11	1.6	13	1.1
2–6 times a week	0	0.0	3	0.9	3	0.6	1	0.2	1	0.1	2	0.2
Once a day or more	0	0.0	0	0.0	0	0.0	0	0.0	4	0.6	4	0.4
**Use of laxatives, etc., to control body weight and shape**												
Never	135	90.0	305	87.1	440	88.0	396	90.2	597	85.8	993	87.5
Once a month or less	7	4.7	22	6.3	29	5.8	24	5.5	66	9.5	90	7.9
2-3 times a month	7	4.7	12	3.4	19	3.8	12	2.7	17	2.4	29	2.6
Once a week	0	0.0	4	1.1	4	0.8	5	1.1	7	1.0	12	1.1
2–6 times a week	1	0.7	5	1.4	6	1.2	1	0.2	5	0.7	6	0.5
Once a day or more	0	0.0	2	0.6	2	0.4	1	0.2	4	0.6	5	0.4
**Exercising for >60 min daily to lose or control weight**												
Never	59	39.3	180	51.4	239	47.8	171	39.0	314	45.1	485	42.7
Once a month or less	20	13.3	68	19.4	88	17.6	68	15.5	180	25.9	248	21.9
2-3 times a month	23	15.3	46	13.1	69	13.8	72	16.4	92	13.2	164	14.4
Once a week	16	10.7	23	6.6	39	7.8	34	7.7	31	4.5	65	5.7

2–6 times a week	24	16.0	30	8.6	54	10.8	82	18.7	66	9.5	148	13.0
Once a day or more	8	5.3	3	0.9	11	2.2	12	2.7	13	1.9	25	2.2
**Weight loss of >9 kg in the last 6 months**												
Never	20	60.6	55	88.7	75	78.9	43	69.4	85	83.3	128	78.0
Once a month or less	8	24.2	6	9.7	14	14.7	14	22.6	6	5.9	20	12.2
2-3 times a month	2	6.1	0	0.0	2	2.1	1	1.6	4	3.9	5	3.0
Once a week	0	0.0	1	1.6	1	1.1	2	3.2	1	1.0	3	1.8
2–6 times a week	0	0.0	0	0.0	0	0.0	1	1.6	1	1.0	2	1.2
Once a day or more	3	9.1	0	0.0	3	3.2	1	1.6	5	4.9	6	3.7
**BMI classification**												
Underweight	0	0.0	48	13.7	48	9.6	0	0.0	37	5.3	37	3.3
Normal	44	29.3	218	62.3	262	52.4	161	36.7	449	64.5	610	53.7
Overweight	74	49.3	43	12.3	117	23.4	207	47.2	165	23.7	372	32.8
Obese	32	21.3	41	11.7	73	14.6	71	16.2	45	6.5	116	10.2
**BMI (kg/m²) ($\bar{X} \pm SD$)**	26.97 ± 4.65		22.45 ± 4.53		23.81 ± 5.01		26.63 ± 3.83		23.71 ± 3.78		24.84 ± 4.05	
